# Understanding the role of traditional healers in the HIV care cascade: Findings from a qualitative study among stakeholders in Mwanza, Tanzania

**DOI:** 10.1371/journal.pgph.0000674

**Published:** 2022-08-15

**Authors:** Dunstan J. Matungwa, Richie Hong, Jeremiah Kidola, Daniel Pungu, Matthew Ponticiello, Robert Peck, Radhika Sundararajan

**Affiliations:** 1 Mwanza Research Centre, National Institute for Medical Research, Mwanza, Tanzania; 2 Department of Anthropology, Rutgers University, New Brunswick, New Jersey, United States of America; 3 Weill Cornell Medical College, New York, New York, United States of America; 4 Department of Emergency Medicine, Weill Cornell Medicine, New York, New York, United States of America; 5 Weill Cornell Center for Global Health, Weill Cornell Medicine, New York, New York, United States of America; 6 Department of Medicine, Weill Bugando School of Medicine, Mwanza, Tanzania; Southern Cross University, AUSTRALIA

## Abstract

Tanzania is HIV-endemic, with 5% prevalence. However, less than half of Tanzanians are aware of their HIV status, and only 75% of adult Tanzanians living with HIV are on antiretroviral therapy (ART). Informal healthcare providers, such as traditional healers, frequently serve as the first line of healthcare in Tanzania. How traditional healers interact with people living with HIV (PLWH) remains unknown. This study sought to understand gaps in HIV care and explore how traditional healers interface with PLWH along the HIV care cascade. We conducted a qualitative study in Mwanza, Tanzania, between November 2019 and May 2020. We invited 15 traditional healers, 15 clients of traditional healers, 15 biomedical healthcare facility staff, and 15 PLWH to participate in a single qualitative interview. Two community focus groups were held with eight male and eight female participants. Participants were 18 years of age or older. Individual experiences with traditional healers and biomedical healthcare facilities, as well as perceptions of traditional healers with respect to HIV care, were explored through interviews. Using a content-analysis approach, codes were grouped into a framework that characterized how traditional healers engage with PLWH throughout the HIV care cascade. PLWH engaged with traditional healers throughout the HIV care cascade, from pre- to post-HIV diagnosis. Traditional healers were described in some cases as facilitating HIV testing, while others were described as delaying testing by providing traditional treatments for HIV symptoms. Traditional medications were frequently used concurrently with ARTs by PLWH. There was concern that healers contributed to ART nonadherence as some PLWH used traditional therapies in search of a “cure” for HIV. Our findings suggest that traditional healers interact with PLWH throughout the HIV care continuum and that collaboration between traditional healers and biomedical healthcare professionals and facilities is needed to improve HIV treatment outcomes.

## Introduction

Human immunodeficiency virus (HIV) is endemic in Tanzania. As of 2018, the country had an HIV prevalence of 5% and approximately 1.4 million people living with HIV (PLWH) [1,2]. The World Health Organization (WHO) and the Tanzanian Ministry of Health advocate for the “test-and-treat” HIV control strategy, which is predicated on access to HIV testing and antiretroviral therapy (ART) treatment to identify and reduce viral loads in PLWH [3]. Despite Tanzania’s high HIV prevalence and the international push to increase HIV testing [4], in 2018 still less than 50% of Tanzanians were aware of their HIV status [2], and only 75% of adult PLWH in Tanzania were on ART [5]. As such, Tanzania—like much of sub-Saharan Africa—is lagging behind the HIV treatment targets set out by the Joint United Nations Program on HIV/AIDS (UNAIDS) to end the HIV epidemic: 95% of PLWH know their HIV status; 95% of people who know their status are on treatment; and 95% of people on treatment have undetectable viral load by 2030 [6]. To achieve these goals, increasing entry in and retention at each step of the HIV care cascade is necessary.

In low resource settings, PLWH face numerous challenges in entering and remaining in HIV care. Previous studies have reported that some of these challenges include shortage of healthcare workers to attend patients, as well as structural barriers to accessing treatment such as long distance to HIV clinical facilities, high transportation costs, and hours of waiting for services [7–9]. In addition, PLWH may avoid clinics due to perceived stigma and discrimination by healthcare workers, as well as a lack of trust in biomedical care [10,11]. These factors have driven disengagement from care and suboptimal adherence to ART regimens [12]. Implementation scientists have advocated for novel, community-based programs as one approach to engaging with PLWH in these settings [13]. However, in order to effectively engage all PLWH, the HIV care cascade must be embedded in socially, culturally, and structurally-informed programs for initiating and maintaining care.

Traditional healers have been proposed as a community-based strategy for enhancing the HIV care cascade [14,15]. Collaboration with traditional healers could be particularly impactful in sub-Saharan Africa where the WHO estimates that 80% of the population seeks their services on a regular basis [16]. Tanzania’s health sector, like in other sub-Saharan African countries, is pluralistic, with people receiving treatment from traditional healers in conjunction with, or in place of, biomedical institutions [17–19].

Evidence suggests that PLWH use the traditional health sector at all stages of the HIV care continuum [20]. However, there is no consensus on how the use of the traditional healthcare impacts engagement with HIV services. According to some studies in Tanzania [21,22], and elsewhere in sub-Saharan Africa [23–30], traditional healers serve as a bottleneck in the HIV care cascade, delaying HIV diagnosis and treatment and decreasing adherence to ART. Other studies found that PLWH who used traditional herbal medicines did so to alleviate medication side effects and did not demonstrate poor ART adherence [31]. Studies have also illustrated that traditional healers provide tailored, individualized care for PLWH [32], which improves the quality of life among their clients living with HIV [18]. The improved quality of life has been attributed to traditional healers’ provision of psychosocial support and a familiar cultural context for healthcare delivery [33]. We, and others, have shown that when trained by healthcare professionals, traditional healers are both willing and able to facilitate HIV testing among their clients [34–36].

Therefore, while there is no clear consensus on how traditional healers impact engagement with the HIV care cascade, a nuanced understanding of these complex relationships is needed to develop interventions to improve uptake of HIV services. The goal of this study was to shed light on this area of controversy through an in-depth examination of how traditional healers engage with PLWH at all stages in the HIV care cascade in an HIV endemic region. We consider the HIV care cascade for PLWH to begin from the pre-diagnosis phase and continue through linkage to HIV clinical care, ART use, and retention in HIV care.

## Methods

### Research design and setting

To collect data from key stakeholders in the HIV care cascade, we conducted a qualitative study that included in-depth interviews and focus group discussions [37]. The study was conducted in Mwanza, a port city on the shore of Lake Victoria, and the second largest city in Tanzania. People in Mwanza and the neighboring regions (of Simiyu, Shinyanga, and Tabora), where the Sukuma and Nyamwezi ethnic groups are dominant, have a long, rich, and popular history of practicing and using traditional and alternative medicine [38,39]. Like in many other parts of the country, people in Mwanza city depend on and use biomedical healthcare services, alternative medicine, and traditional medicine [17–19]. The city also has public and private biomedical clinics providing free HIV testing, among other services. Traditional healers—herbalists, birth attendants, spiritualists, and bone setters [40]—are found throughout the city and across villages. Traditional healers’ operations in Tanzania are overseen and regulated by the Traditional and Alternative Medicines Act (No. 23 of 2002) [41]. One of the key provisions of this law is that every traditional healer must be formally registered with the Traditional and Alternative Health Practice Council under the Ministry of Health [41].

### Sampling and recruitment

Using purposive sampling, we identified and recruited participants from five key stakeholder groups in the HIV care cascade: (1) HIV clinic staff, (2) PLWH, (3) traditional healers, (4) adult people receiving care from traditional healers, and (5) adult community members (women and men). Eligible participants were aged ≥18 years, members of one of the five aforementioned stakeholder groups who provided a written informed consent, and agreed to be audio-recorded. With the assistance of clinicians in charge, HIV clinic staff—medical doctors, nurses, and pharmacists (*n* = 15)—were identified and recruited from five HIV clinics in Mwanza city. PLWH (*n* = 15), like HIV clinic staff, were also identified and recruited at the same five HIV clinics. Traditional healers (*n* = 15) were identified from a list of registered and currently practicing healers from the Mwanza Regional Traditional and Alternative Medicine Coordinator. Traditional healers were contacted by phone and invited to take part in the study. Adults receiving treatment or care from traditional healers (*n* = 15) were identified and recruited at the traditional healers’ delivery facilities following completion of their treatment or care. We recruited in this manner to avoid the impression that participating in the study was a condition for receiving treatment or care from the traditional healer.

The sample size was set at 15 interviews per group for HIV clinic staff, PLWH, traditional healers, and adults receiving care from traditional healers. Previous studies show that, within homogenous groups, a sample size of nine interviewees is adequate to achieve code saturation [42]. A total of 60 semi-structured, one-hour interviews were conducted. Adult community members (eight men and eight women) were recruited from a single community on the outskirts of Mwanza city—with the help of the community leaders—to participate in two focus groups. Lasting approximately one hour, focus group discussions were intended to elucidate an understanding of common community perspectives on traditional healing practices and HIV. Focus group discussion guides followed a similar structure as the individual interview guides. Each group consisted of eight participants of the same gender. We conducted two focus groups because prior research has shown that two to three focus groups are sufficient for generating and identifying meaningful themes [43].

### Data collection

Between December 2019 and June 2020, stakeholders were invited to participate in either a single semi-structured interview or a focus group discussion. All invitees agreed to participate. Four Tanzanian research assistants (two males and two females) and a study coordinator (co-author DP)—all fluent in Kiswahili (the Tanzanian national language) and English—conducted interviews and focus groups. Prior to data collection, the research assistants and the study coordinator were oriented to the research question and study objectives, as well as given a refresher training on how to collect qualitative data using semi-structured interview and focus group discussion guides.

Interviews and focus groups were audio recorded and conducted in Kiswahili in a private location obtained at or around the site where participants were recruited. Semi-structured guides were used to ensure that topics were consistent across interviews and focus groups (S1 Text). The main interview topic was to understand how traditional healers engage with PLWH prior to and after HIV diagnosis. Before fieldwork, semi-structured guides were pilot tested with one member of each stakeholder group. Data collected during the pilot test were excluded from the study dataset.

### Data management and analysis

Interviews and focus group discussion transcripts were analyzed as part of a single dataset. Professional Tanzanian translators fluent in both Swahili and English transcribed the audio recordings verbatim in Kiswahili, then into English for analysis. Author DJM reviewed all Kiswahili to English transcripts for quality and translational integrity. Three authors (RH, MP and RS) independently reviewed the transcripts and created a coding scheme relevant to traditional healers’ engagement with PLWH prior to and following HIV diagnosis. A phenomenological framework was used for data analysis as we sought to understand participant perspectives and experiences within the contexts of their own worldviews [44,45]. Codes were produced using an open-coding approach and refined through constant comparison [46]. The three authors discussed and agreed on the final coding scheme. Final codes were grouped into themes and analyzed using content analysis approach [47]. Finally, representative quotations were selected to illustrate the study findings.

The study team used diverse strategies to ensure the trustworthiness of this qualitative study across the quality criteria in qualitative research: credibility, transferability, dependability, confirmability, and reflexivity [48,49]. Credibility was ensured by using three strategies: prolonged engagement, triangulation, and persistent observation. With regard to prolonged engagement, the study coordinator and the research assistants asked the participants the main and follow-up questions and encouraged them to support their responses with concrete examples of situations they were describing. With regard to triangulation, data for this study were collected from diverse groups of stakeholders (data triangulation), using interviews and focus group discussions (method triangulation), and were coded, analyzed and interpreted by authors RH, MP, and RS (investigator triangulation). And with regard to persistent observation, authors RH, MP, and RS read and re-read the data transcripts to learn about how traditional healers engage with PLWH before and after HIV diagnosis.

To enable the transferability of this study, we have described the study site and setting in Mwanza city, Tanzania, where use of traditional medicine is in common despite the presence of modern health facilities. We have also described the study participants’ demographic features, sampling procedure, recruitment strategies, inclusion and exclusion criteria, and the sample size. Dependability and confirmability of findings were also ensured by having three authors (RH, MP, and RS) code the data. Thus, themes emerging from the data were identified by separate authors and endorsed by the three authors through consensus. Lastly, an iterative analytical strategy allowing for the reflexive evaluation of data was followed, where authors acknowledged and challenged the role of one another’s positionality in data coding, analysis, and interpretation [48,49].

### Research ethics approvals

This study received ethics approvals from the Medical Research Coordinating Committee (MRCC) in Tanzania (Protocol no. NIMR/HQ/R.8a/Vol.IX/2136) and Weill Cornell Medicine (Protocol no. 19–04020274). This work was funded by the Weill Cornell Kellen Faculty Fellowship. Funders had no role in the research design or decision to publish this work. Clinicians in charge of HIV clinics and traditional healers provided consent for recruiting patients at their clinics. All participants provided a written consent to participate in the interviews or focus groups. A copy of the signed consent form was given to the participant for their records. All participants received de-identified study numbers to maximize confidentiality. Since the nature of the focus groups impedes researchers from guaranteeing complete confidentiality (as some participants may disclose the content of the interview or focus group with non-participants), participants were urged to respect fellow participants’ privacy and to not share the content of the discussions with non-participants [44]. Each participant received 10,000 Tanzanian Shillings (~4 USD) as a compensation for their time to participate.

## Results

### Characteristics of study participants

Overall, 60 semi-structured interviews were conducted among HIV clinic staff (*n* = 15), PLWH (*n* = 15), traditional healers (*n* = 15), and clients of traditional healers (n = 15). Two focus groups were conducted with community members: adult men (*n* = 8) and women (*n* = 8). Summary characteristics of participants are shown in Table 1. The majority of participants were male (*n* = 42, 55.7%). Traditional healers were older than their patients, HIV clinic staff, and PLWH. Most traditional healers and their clients had primary or secondary school education while most of HIV clinic staff had at least a post-secondary school education certificate (required for positions such as nursing jobs).

**Table 1:** Summary characteristics of study participants.

Characteristics	Traditional Healers (*n* = 15)	Clients of Traditional Healers (*n* = 15)	HIV clinic staff (*n* = 15)	PLWH (*n* = 15)	Male focus group (*n* = 8)	Female focus group (*n* = 8)
**Female**	3 (20%)	5 (33.3%)	10 (66.7%)	8 (53.3%)	0 (100%)	8 (100%)
**Age in years, median (IQR)**	50 (42–64)	36 (29–45)	47 (30–53)	47 (40–55)	28 (26–41)	35.5 (27.5–44.5)
**Highest level of education**	Primary, 9 (60%) Secondary, 6 (40%)	None, 1 (6.7%) Primary, 7 (46.7%) Secondary, 4 (26.7%) Diploma, 1 (6.7%) University, 2 (13.3%)	Certificate, 8 (53.3%) Diploma, 5 (33.3%) University, 2 (13.3) %	None, 1 (6.7%) Primary, 5 (33.3%) Secondary, 8 (53.3%) Diploma, 1 (6.7%)	Primary, 2 (25%) Secondary, 3 (37.5%) Certificate, 1 (12.5%) Diploma, 2 (25%)	Primary, 5 (62.5) Secondary, 2 (25) Certificate, 1 (12.5)
**Occupation**	Traditional healer, 15 (100%)	Business owner, 4 (26.7%) Skilled laborer, 7 (46.7%) Unskilled laborer, 1 (6.7%) Unemployed, 1 (6.7%)	Nurse, 13 (86.7%) Pharmacist, 1 (6.7%) Medical doctor, 1 (6.7%)	Business owner, 10 (66.6%) Skilled laborer, 1 (6.7%) Unskilled laborer, 3 (20%) Unemployed, 1 (6.7%)	Business owner, 4 (50%) Skilled laborer 2 (25%) Unskilled laborer 2 (25%)	Business owner, 4 (50%) Skilled laborer, 2 (25%) Unskilled laborer, 2 (25%)

### Overview of the results

**Fig 1:**
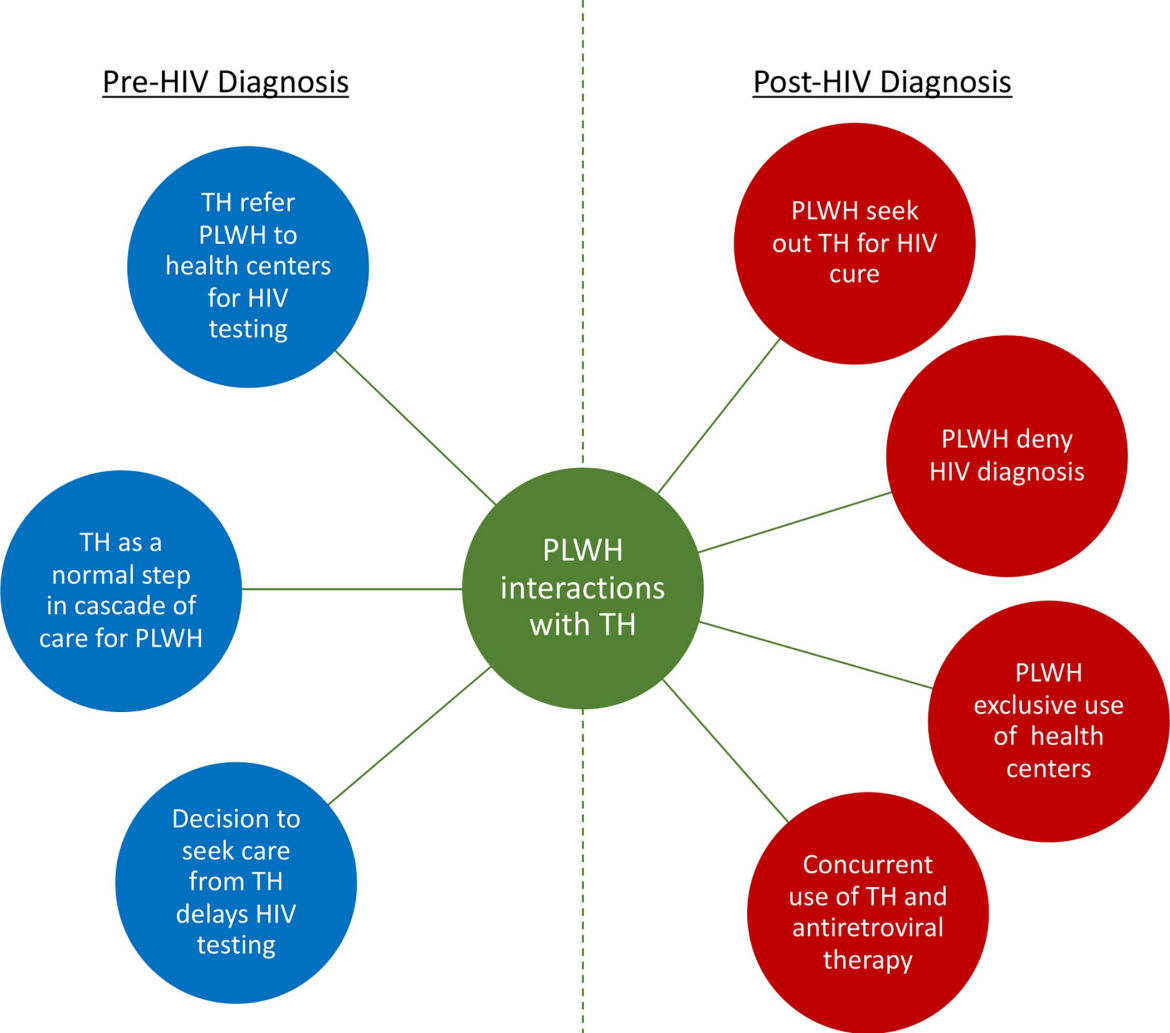
Schematic representation of traditional healers’ (TH) engagement with people living with HIV (PLWH) before and after HIV diagnosis.

We categorize traditional healers’ engagement with PLWH in two periods—prior to and following HIV diagnosis—and describe the variety of forms it takes in each period. Our findings show that, before HIV diagnosis, traditional healers’ engagement with PLWH takes three forms: (1) traditional healers have knowledge of HIV and sometimes refer PLWH for voluntary HIV testing and counseling at HIV clinic facilities, (2) consulting traditional healers is a regular step in the care cascade for PLWH, and (3) patients’ decision to attend traditional healers delays HIV testing. After HIV diagnosis, traditional healers’ engagement with PLWH takes four forms: (1) PLWH seek out traditional medication for HIV cure, (2) PLWH deny HIV diagnosis and return to traditional healers for continued treatments, (3) PLWH stop visiting traditional healers and exclusively use biomedical healthcare services, and (4) PLWH use traditional treatment and ART concurrently. This figure summarizes traditional healers’ engagement with PLWH before and after HIV diagnosis (Fig 1).

### Traditional healers engage with PLWH prior to HIV diagnosis

Based on participants’ narratives, traditional healers’ engagement with PLWH prior to HIV diagnosis takes three forms. First, traditional healers refer their clients to healthcare facilities for HIV testing. Participants described that most traditional healers are knowledgeable about HIV symptoms and that when they observe such symptoms in their clients, they refer them for HIV testing. In this way, rather than being the barrier, traditional healers facilitate HIV diagnosis, the first and crucial step in the HIV care cascade.

“*I have heard some saying that their traditional healers told them to first get tested because there are some [traditional healers] who might see certain symptoms and think that there is a possibility that the patient is HIV positive*.” (HIV clinic staff, Medical doctor, Male, 31 years old).

Second, participants reported that people, irrespective of their HIV status, regularly seek treatment and care from traditional healers. Healthcare facility staff were familiar with this process, and they regarded it as a normal part of healthcare seeking, as most people in Tanzania utilize multiple therapeutic modalities to manage their health.

“*[Traditional healers] will list a number of diseases such as leg pain*, *stomach problems*, *and other diseases that they can help with*. *Things like fertility issues*, *some say they can treat that*. *You sometimes hear that traditional healers can treat 99 diseases except one*. *What is that one disease is that they cannot treat*? *HIV*.” (HIV clinic staff, Nurse, Male 27 years old).

Third, participants reported that, in some cases, treatment and care seeking from traditional healers creates delays to HIV testing and diagnosis. On the one hand, this happens because patients and their families believe that certain symptoms reflect traditional illnesses, and therefore require traditional treatments. Some participants reflected on how beliefs in the power of traditional and faith healing practices delays HIV testing and diagnosis.

“*But there are families who believe very much in traditional things*. *They believe they have been bewitched*. *There are also those who believe that God heals so they go to churches to be prayed for*, *[while] others go to traditional healers*…*I have a friend who suffered a lot [seeking these types of treatments]*, *but at the end of the day she came to find out that she was HIV positive*.” (Community focus group participant, Male, 37 years old).

“*During his time as a welder*, *he had some affairs through which he contracted HIV*. *He started to get sick*, *and he was sick for a while*. *He started saying that people were jealous of him because he had built a house and so he started visiting traditional healers…he had become stick thin and they did not know what he was suffering from but traditional healers were saying that he was getting better*. *They would go from one traditional healer to the next… but to no avail*, *he did not get better through traditional treatment*.” (HIV clinic staff, Nurse, Female, 57 years old).

Our data also indicate that delays to HIV testing and diagnosis occur because HIV is outside the purview of traditional healing, and in some cases, healers do not recognize its signs and symptoms. For example, one PLWH explained that his HIV symptoms were initially misinterpreted by the traditional healer as yellow fever:

“*By the time I went to the hospital*, *I had faced a lot of loss*, *because before going to the hospital*, *I used traditional medication to treat the illness*, *which at the time*, *was not diagnosed and was instead thought to be yellow fever*.” (PLWH, Male, 53 years old).

Prolonged traditional treatment bolsters delays in HIV testing and diagnosis, which in some cases can lead to suffering and death. Another participant described the experience of a friend as follows:

“*When he went for traditional treatment*, *he was given herbs for treating diarrhea and vomiting… but this made it worse*. *When we took him to the healthcare facility*, *it was already too late*, *and he lost his life*… *The traditional healer said that the patient had swallowed some things and so he had to get rid of them through diarrhea and vomiting*, *while the truth was that he was HIV infected*.” (PLWH, Male, 47 years old).

### Traditional healers engage with PLWH following HIV diagnosis

Based on participants’ narratives, we found that traditional healers’ engagement with PLWH after HIV diagnosis takes four forms. First, PLWH may visit traditional healers in pursuit of a cure to HIV because of the belief in the preternatural etiology of their HIV infection or increased efficacy of traditional medicine. A HIV clinic nurse described the experience of a patient who sought a cure from a traditional healer:

“*She decided to go to a traditional healer and when she got there the doctor told her that someone had thrown an HIV demon at her*, *and that after the doctor gave her a certain medicine then the demon would vacate her body*. *That woman came here believing that she was no longer HIV positive after her visit to the traditional doctor… We tested her again*, *but the result was still positive*.” (HIV clinic staff, Nurse, Male, 29 years old).

In another case, a PLWH disengaged from HIV care after visiting a healer who promised a cure for HIV, and he believed that he no longer needed to continue ART:

“*I know a client of ours who was HIV positive*. *He had a relative who convinced him to go to a traditional healer who cures the disease*… *In the end*, *we told him the door [at the clinic] is open*, *in case things did not go well he would be welcome*. *After nine months*, *he came back really ill*. *He was not doing well*.” (HIV clinic staff, Nurse, Female, 55 years old).

In some cases, following a new diagnosis PLWH may deny their status and seek care from traditional healers instead of health facilities. Respondents reported that PLWH may deny their HIV diagnosis because they believe that they are in fact suffering from a spiritual illness.

“*I think many people tend to deny they have HIV and say it is a spiritual illness*, *and maybe and that so-and-so can treat them*. *They try to seek other traditional treatment [instead of ART]*.” (Traditional healer, Male, 85 years old).

Some PLWH may also mistrust biomedicine in general, so they do not accept the results of the HIV tests and recommendations provided by HIV clinic staff. Instead, they may hope that symptoms could be attributed to “traditional” etiologies, and thereby cured via traditional therapies.

“*Because they do not believe in [HIV] tests in the first place*, *they go to see a traditional healer … It is not easy for them to have faith in [the HIV testing]*. *There are some who–after testing positive for HIV–would be in denial and decide to go to a traditional healer saying they have been bewitched*.” (PLWH, Female, 59 years old).

Third, in some cases after HIV diagnosis, some PLWH choose to use biomedical care exclusively without interacting with traditional healers again.

“*After getting tested here in Bugando where I found out I was infected*, *I took immediate steps to join and start using medicine [ART]*. *I did not go back to a traditional healer*, *and I did not use any traditional medicine*. *I started [in HIV care] here immediately*.” (PLWH, Male, 43 years old).

Finally, following HIV diagnosis, some PLWH may receive concurrent treatment and care from both healthcare facilities and traditional healers. Based on the participants’ narratives, two underlying factors drive this phenomenon. The first factor is that some PLWH on ART use traditional medicine as way to gain relief from HIV symptoms or ART side effects.

“*There are patients who are open with their status and are on ARTs that come asking for help*. *I am clear with them that there is no treatment [for HIV] but there is medication to boost their immunity that I can offer*.” (Traditional healer, Male, 46 years old).

*Interviewer*: “*What illnesses or health issues do you think a traditional healer can treat*?”

*Respondent*: “*Even with HIV*, *they can help in symptom reduction*.” (Client of traditional healer, Female, 45 years old).

The second factor is that other PLWH on ART receive care from traditional healers for psychosocial support regarding their HIV diagnosis and care. This psychosocial support helps PLWH, among other things, with ART adherence and remaining in HIV care.

“*[My client] was afraid*, *so I took him myself to the hospital for HIV testing and he turned out to be positive*. *On confirmation of HIV status*, *the hospital staff asked to take over*. *I mentioned since he was my patient*, *I should continue working with him*. *We went back to my clinic*, *and I advised him about starting medication [ART] to increase his CD4 count*, *which he agreed to do*. *He is still on medication*, *and we are in contact*.” (Traditional Healer, Male, 46 years old).

“*I will [support patients in HIV care]*. *If you just let the patient walk away*, *then you will have killed them*. *But*, *if you keep helping the patient*, *you will help them have faith in you so that they can continue to use ART*.” (Traditional healer, Male, 42 years old).

## Discussion

Our findings clarify how traditional healers impact the HIV care cascade, showing that PLWH interact with traditional healers throughout the entire cascade. Prior to HIV diagnosis, traditional healers (1) assist in the identification of HIV symptoms and encourage their clients to get tested, (2) are a normal step in the HIV care cascade, and neither facilitate nor delay HIV testing, and (3) provide traditional therapies for the treatment of their patients’ symptoms. We found that patients and their families make the decision to pursue traditional therapies for illness symptoms, which can delay HIV testing and care, while in other cases healers failed to recognize signs and symptoms of HIV infection. After HIV diagnosis, traditional healers (1) could siphon patients away from biomedical treatment by promising a cure for HIV or providing treatment to PLWH denying their diagnosis, (2) may not receive PLWH, as the latter prefer exclusive use of biomedical facilities for HIV care, (3) may offer traditional treatment concurrently with ART; and (4) provide psychosocial support for PLWH to remain in HIV care. Traditional healers have the potential to impact engagement with HIV care in many different ways including facilitating engagement with HIV services.

Our findings point to a chasm between healthcare facilities’ staff perceptions and the reported lived experiences of HIV patients who receive care from traditional healers. Healthcare facility staff frequently reported that traditional healers act as a barrier to HIV testing and HIV medication adherence, with anecdotes of patients arriving at healthcare facilities in late-stage HIV with poor outcomes. Others shared stories they had heard from their colleagues, although they had no personal experience working with such patients. This tendency shifts blame to traditional healers for delaying care and contributing to morbidity and mortality [21]. Conversely, our data suggests that traditional healers do not instruct patients to avoid HIV testing or treatment. Instead, individual patients—often in consultation with some of their family members—voluntarily choose traditional medicine to treat their ailments.

Staff at healthcare facilities also expressed concerns that traditional healers could persuade PLWH to disengage with ART by purporting to cure HIV. We also noted accounts of PLWH using traditional medicine for treatment and care while denying their HIV diagnosis, even though no traditional healer participating in this study endorsed having a cure for HIV. The driver of ART disengagement in these cases appears to lie with the individual PLWH and their families who choose where and how to receive treatment and care. The experiences of the healthcare facility staff contrast with traditional healers, who all reported that HIV required biomedical diagnosis and treatment and consequently, referred clients to healthcare facilities to receive testing. Our findings are consistent with previous studies, which found that 75% of Tanzanian traditional healers recognized HIV symptoms and appropriately referred patients to biomedical care [50]. However, we note accounts from PLWH demonstrating that HIV is outside of the purview of traditional healing, and HIV symptoms have gone unrecognized. In Tanzania, traditional healers do not receive formal training or education on recognizing HIV symptoms, and they are not part of community HIV control programs.

Many PLWH reported exclusive healthcare facility utilization following HIV diagnosis, opting not to return to traditional healers. In contrast, traditional healers reported that they often provide supportive care for PLWH and that concurrent use of traditional medicine along with ART is common. Taken in conjunction with prior studies that have shown that patients do not disclose their use of traditional medications to biomedical facilities, lack of disclosure may be attributed to the stigma that healthcare facility staff have against traditional healers, or the fact that healthcare facility staff do not ask their patients if they seek out treatment and care from traditional healers [51]. Other studies have demonstrated similar negative perspectives of traditional medicine among biomedical healthcare facility staff who voice concerns with unstandardized treatments and possible drug interactions when used with biomedical medications [21]. The perspective that biomedicine and traditional medicine are incompatible may actually fuel the siphoning of patients away from biomedical healthcare facilities, as PLWH may feel compelled to choose one modality of treatment over another. In many cases, traditional healers may be preferred as they are more accessible in communities compared with biomedical healthcare facilities, where HIV clinical treatment facilities carry stigma [50]. We and others have also shown that patients choose to receive care from traditional healers instead of medical clinics due to trusting relationships with healers and the perception that healers provide more psychosocial support [34,36,52–54]. Modern medicine has also been criticized for failing to resolve all health problems, and thus, traditional medicine promises to fill the void in the biomedicine health care system [51].

Given that traditional healers interface with PLWH throughout the HIV care cascade, some have proposed formally integrating traditional healers into the HIV control programs [55–58]. Our data from all stakeholder groups, including healthcare facility staff, largely agree that improving HIV treatment and care for PLWH would require a functional collaboration between traditional healers and biomedical healthcare professionals and facilities. Healthcare facility staff and PLWH all recognize the prominent role traditional healers play in their communities. Because they are integrated and accessible in communities, traditional healers are able to reach a wider range of individuals than biomedical facilities. As such, traditional healers may be “trusted messengers” in their communities who could improve entry and retention in the HIV care cascade [59]. The current lack of collaboration between traditional healers and the biomedical system create spaces for PLWH to fall through the cracks and not get tested or treated for HIV [35]. Consequently, HIV service delivery in Tanzania could benefit from a formal collaboration between traditional healers and clinic-based medical providers.

This study has some limitations. First, we did not speak with any PLWH receiving care at traditional healers’ delivery facilities, and therefore may not have captured the perspectives of PLWH who are not in HIV care. Second, the sample of medical doctors among healthcare facility staff that we worked with is small. We therefore might have missed ideas from this important group in the HIV care cascade. Third, only healers registered with the Traditional and Alternative Health Practice Council were included in this study. The views of those healers who have not registered may vary from those registered. We also acknowledge that social desirability bias may have impacted participant responses. It is possible that healers and PLWH may have perceived interviewers as connected with biomedical care/providers, and therefore been inclined to endorse a strictly biomedical perspective on HIV. Finally, qualitative data are highly contextual, and therefore, further research is needed to evaluate if our findings are transferable to other contexts where traditional healers are commonly utilized.

## Conclusion

Traditional healers engage with PLWH throughout the HIV care cascade. For medically pluralistic regions such as Tanzania where people, regardless of their HIV status, use both traditional medicine and biomedicine, we should consider formally integrating traditional healers into HIV treatment and care as partners throughout the cascade. However, there is need for further studies to establish how best this integration can be practically implemented. In the meantime, there is a critical need for improving communication between healthcare facility staff and traditional healers to better serve PLWH. The differences in attitudes and beliefs shared between the healthcare facility staff, traditional healers, and PLWH may be driving forces behind why a formal, functional collaboration between traditional healers and healthcare facility staff has been elusive. As such, PLWH can fall through the cracks of a fragmented healthcare system and either fail to get diagnosed or treated. If traditional healers and healthcare facility staff can better understand each other, communicate, and work in solidarity, we will significantly improve PLWH treatment and outcomes.

## Supporting information

S1 Checklist COREQ checklist. (PDF)

S1 Text Focus group discussion and interview guides for participants. (DOCX)

S2 Text Inclusivity in global research. (DOCX)
