# Supplementary material for: Understanding the role of traditional healers in the HIV care cascade: Findings from a qualitative study among stakeholders in Mwanza, Tanzania
Source: PLOS Glob Public Health. 2022 Aug 15;2(8):e0000674. doi: 10.1371/journal.pgph.0000674 (PMC10021224; doi:10.1371/journal.pgph.0000674)
Supplement: S1 Text — (DOCX) [file pgph.0000674.s002.docx]

**S1 Text:** Interview and focus group guides

**A. Focus Group Discussion guide for community members:**

Questions about community health resource utilization

1. When people become sick in your communities, please describe how they proceed to receive care for their symptoms? [For example, for fevers or headaches?]

- prompts: What resources are commonly utilized first? Discuss perceived effectiveness of healthcare treatments and providers? Describe what happens if initial treatment is not successful.

2. Let’s talk about hospitals and clinics generally. What is your opinion of these healthcare services?

- prompts: Discuss providers trustworthiness and effectiveness. Explore accessibility of services. Consider costs and wait time. Probe for negative or positive opinions including personal experiences.

Questions regarding traditional medicine and healers

1. Can you tell me what you know about traditional healers?

- prompts: Describe what types of problems or illnesses healers can address?

2. What are your thoughts about the services provided by traditional healers?

- prompts: Discuss healers’ capacity to help their patients, trustworthiness, effectiveness. Explore accessibility of services. Probe for negative or positive opinions, personal experiences with traditional medicine (rumors, stories, etc).

3. Have you ever personally utilized a healer, or taken a family member to a healer?

- prompts: what kind of healer, what were the symptoms, was the treatment effective? What happened next?

Questions on existing HIV testing and care

1. Please tell me your opinion about the current availability of HIV services for our local communities.

- prompts: Do people engage with resources? Why/Why not? Are testing options and locations sufficient?

2. What are the biggest barriers to people receiving HIV testing and care, in your opinion?

- prompts: Are there any ways that the HIV care could be improved, in your opinion? Ease of access, confidentiality, wait time?

**B. Interview Guide for clients of traditional healers:**

Questions on Traditional Healer utilization

1. You recently completed a visit with a healer. Can you tell me about why you came to see the healer today?

- prompts: what was the problem? what kind of treatment did you receive? Have you previously been elsewhere to receive treatment for this problem?

2. What are your thoughts about the services provided by traditional healers?

- prompts: Discuss healers’ capacity to help their patients, trustworthiness, effectiveness. Explore accessibility of services.

3. What kinds of problems do you think Traditional Healers can address?

- prompts: which diseases, physical ailments, spiritual ailments?

4. What do you think of people who *never* visit healers?

- prompts: do you know anyone like this? What do you think of that decision?

Questions on prior experiences with Biomedicine

1. Have you ever been seen at a hospital or clinic? [If yes] Please tell me about the last time you went to a hospital or clinic for treatment.

- prompts: what were your symptoms? what treatment(s) did you receive? Were you healed? What was your overall experience (good or bad)?

- [If no] Please tell me more about your decision not to visit hospitals or clinics (then skip to Question 3).

2. Please tell me about how you arrived at the clinic, and your experience of receiving your treatment/services.

- prompts: how did the patient obtain money for transport, cost of visit, medication/prescription? Explore what duties were missed in order to obtain treatment, such as work or school

3. Let’s talk about hospitals and clinics generally. What is your opinion of these healthcare services?

- prompts: Discuss provider competence, trustworthiness, effectiveness. Explore accessibility of services.

Questions on HIV testing

1. Can you please tell me about the last time you received an HIV test?

- prompts: where and when was it received? What caused you to undergo testing? were you embarrassed? Nervous to be recognized? Did you wait a long time? Did you trust the counselor? Do you trust the results?

[if never received one, please tell me about your decision to not test.]

2. Please tell me about the pre- and post-test HIV counseling you received.

- prompts: How was it helpful, supportive, informative? Describe how can HIV counseling be improved.

**C. Interview guide for HIV clinic patients**

Questions on prior experiences with Biomedicine

1. You recently completed a visit at the HIV clinic. Can you tell me about why you came here today?

- prompts: what treatment(s) did you receive? What was your overall experience (good or bad), and why?

2. Please tell me about how you arrived at the clinic, and your experience of receiving your treatment/services.

- prompts: how did the patient obtain money for transport, cost of visit, medication/prescription? Explore what duties were missed in order to obtain treatment, such as work or school?

3. Let’s talk about hospitals and clinics generally. What is your opinion of these healthcare services?

- prompts: Discuss provider competence, trustworthiness, effectiveness. Explore accessibility of services.

Questions on Traditional Healer utilization

1. Have you ever visited a traditional healer for treatment?

- prompts: what was the problem? what kind of treatment did you receive? Have you previously been elsewhere to receive treatment for this problem?

2. What kinds of problems do you think Traditional Healers can address?

- prompts: physical ailments, spiritual ailments? Have they ever helped you or your family?

3. What do you think of people who prefer healers over clinics or hospitals?

- prompts: do you know anyone like this? What do you think of that decision?

Questions about HIV care cascade

1. Please tell me about your experiences with HIV testing.

- prompts: how and when did you test HIV-positive? What was your experience receiving that test? What symptoms motivated testing? How were your results handled by the clinic and counselors?

2. Please tell me about your experiences receiving HIV care at this clinic.

- prompts: Describe your relationship with your HIV counselor. How do they support you to adhere to medication, and come to your follow up visits?

**D. Interview guide for traditional healers**

Questions about healer versus biomedical utilization

1. Please tell me about your most recent visit to a biomedical facility (clinic or hospital).

- prompts: What symptoms or problems stimulated the visit? What treatments or advice were given?

2. What is your opinion of biomedicine in general, and why?

- prompts: Discuss provider competence, trustworthiness, effectiveness. Explore accessibility of services.

Questions regarding use of HIV services

1. Have you ever received an HIV test? *If yes*, please tell me about the last time you received an HIV test?

- prompts: where and when was it received? What caused you to undergo testing? if never received one, please tell me about your decision to not test

2. Please tell me what you know about HIV.

- prompts: what are common symptoms? How is it acquired? How is it diagnosed and how is it treated? Have you ever had a client with HIV? *If yes*, what kind of service did you provide?

3. Have you ever referred any clients to the HIV clinic for testing?

*- If yes*, please tell me about that experience. What symptoms did the patient have? How did the referral work? How was the patient received? Did you feel your concern was respected by the HIV clinic?

4. Are there any ways that the HIV testing process can have been improved, in your opinion?

- prompts: ease of access, confidentiality, wait time, trustworthiness of results?

5. Can HIV clinicians and traditional healers work together to help patients who need HIV testing?

- prompts: If yes, how might this work? If no, why not?

**E. Interview guide for HIV clinic staff**

Questions about community-based HIV testing

1. Please tell me your opinion about the current availability of HIV testing for our local communities.

- prompts: Do people engage with resources? Why/Why not? Are testing options and locations sufficient?

2. What are the biggest barriers to people receiving HIV testing, in your opinion?

- prompts: Please describe how the HIV testing process could be improved, in your opinion? Consider accessibility, confidentiality, wait time.

Questions regarding traditional medicine and healers

1. Can you tell me what you know about traditional healers?

- prompts: Which types of healers are you familiar with? what do they practice?

2. What do you think of traditional healing?

- prompts: negative or positive opinions, personal experiences with traditional medicine, rumors, stories, etc.

3. Have you ever personally utilized a healer, or taken a family member to a healer?

- prompts: what kind of healer, what were the symptoms, was the treatment effective? What happened next?

4. Do you think your clinic patients have used traditional healing? Why or why not?

- prompts: Is traditional medicine use considered common, or rare among PLHIV?

5. How do you feel when patients tell you they have received traditional healing before discovering they are HIV positive?

- prompts: Tell me about an instance with a patient who had such an experience. How did you react? What is your opinion of the healer in that case?

Questions exploring collaborating with healers

1. Do you think that traditional healers can help to support patients undergo HIV testing? Why/why not?

- prompts: Do they worry about HIV? Do you think they recommend HIV testing for patients? What motivates the care they provide?

2. Can HIV clinicians and traditional healers work together to help patients who need HIV testing?

- prompts: If yes, how might this work? If no, why not?
